# Are Patients with Psoriasis and Psoriatic Arthritis Undertreated? A Population-Based Study from Southern Italy

**DOI:** 10.3390/jcm10153431

**Published:** 2021-07-31

**Authors:** Ylenia Ingrasciotta, Valentina Isgrò, Valentina Ientile, Michele Tari, Gianluca Trifirò, Claudio Guarneri

**Affiliations:** 1 Department of Biomedical and Dental Sciences and Morphofunctional Imaging, University of Messina, 98125 Messina, Italy; vientile@unime.it; 2 Department of Diagnostic and Public Health, University of Verona, 37134 Verona, Italy; valentina.isgro@univr.it (V.I.); gianluca.trifiro@univr.it (G.T.); 3 Caserta-1 Local Health Service, 81100 Caserta, Italy; michele.tari@aslcaserta1.it; 4 Department of Biomedical and Dental Sciences and Morphofunctional Imaging, Section of Dermatology, University of Messina, 98125 Messina, Italy; claudio.guarneri@unime.it

**Keywords:** undertreatment, biological drugs, psoriasis, psoriatic arthritis

## Abstract

This study aimed to explore the pattern of use of different treatment lines in psoriasis (PsO) and psoriatic arthritis (PsA) patients from Southern Italy. A retrospective cohort study was performed during the years 2010–2018 using data from the Caserta Local Health Unit (LHU) claims database. All of the PsO or PsA patients were identified. The proportion of PsO/PsA patients untreated or treated with ≥1 drug classes (i.e., non-disease-modifying antirheumatic drugs (non-DMARDs), conventional synthetic DMARDs (csDMARDs), biological drugs (bDMARDs) or targeted synthetic small molecules (tsDMARDs)) was calculated in the years 2016–2018. Among the bDMARD users, the median times from the first registered PsO/PsA diagnosis/from the first csDMARD to the first bDMARD were calculated. Overall, 10,296 (1.1%) and 1724 (0.2%) PsO and PsA patients were identified. More than half of the PsO patients (*N* = 5301; 51.6%) and 15% of the PsA patients (*N* = 251) were not treated with any drug. A very low proportion of PsO patients (*N* = 121; 1.2%) received csDMARDs/bDMARDs dispensing. Instead, 538 (32.2%) PsA patients were treated with bDMARDs. The median times from the first diagnosis to the first bDMARD dispensing were 54.0 (Q1–Q3: 30.5–72.2) and 13.3 (Q1–Q3: 3.1–43.9) months in the PsO and PsA patients, respectively. The median time from the first csDMARD to the first bDMARD dispensing was shorter in the PsO [9.2 months (Q1–Q3: 5.5–30.0)] than in the PsA [14.5 months (Q1–Q3: 8.6–33.5)] patients. A potential undertreatment of PsO (much less for PsA) in an LHU from Southern Italy, with a particularly low use of more recently marketed drugs, such as biological ones, was shown.

## 1. Introduction

Psoriasis (PsO) is a chronic, inflammatory skin condition associated with significant morbidity and mortality [1]. The estimated prevalence of PsO in Italy is 2.7%, with no significant difference according to sex (3.0 in females vs. 2.7 in males), the while incidence was reported as 2.3/1000 person-years [2,3,4]. About 20–30% of the patients with PsO suffer from psoriatic arthritis (PsA) [5,6], which can further deteriorate quality of life by affecting physical function [7,8].

Various topical medications, phototherapy and systemic medications are available to treat patients with PsO, based on disease severity. Topical treatments represent the cornerstone of treatment for mild-to-moderate PsO: topical corticosteroids (e.g., betamethasone, hydrocortisone, methylprednisolone) and vitamin D analogues (calcipotriene, calcipotriol and calcitriol) are first-line therapies, and all of the other systemic drugs are often used with concomitant topical therapy [9,10,11,12]. For the PsA treatment, nonsteroidal anti-inflammatory agents (NSAIDs) can be used as a symptomatic treatment, while glucocorticoids should be used only as a relief treatment at the lowest dose and for the shortest time duration [13]. For patients with moderate to severe PsO/PsA diseases, systemic pharmacological and non-pharmacological treatments should be considered. The approved systemic therapies for PsO in Italy include phototherapy and photochemotherapy (i.e., psoralen plus UVA light (PUVA)) as well as pharmacological options such as cyclosporine, methotrexate (MTX), acitretin (first-line systemic agents), apremilast (the first selective inhibitor of phosphodiesterase 4 (PDE4)) and biological drugs [9,11,12].

According to the Group for the Research and Assessment of Psoriasis and Psoriatic Arthritis (GRAPPA) [14] and the European League Against Rheumatism (EULAR) recommendations [15], the traditional systemic agents (i.e., MTX, cyclosporine, acitretin, leflunomide, sulfasalazine) are grouped as conventional synthetic disease-modifying antirheumatic drugs (csDMARDs). Among the biological drugs (named as “bDMARDs”), some tumour necrosis factor (TNF)-alpha inhibitors (etanercept, infliximab and adalimumab) and some interleukin (IL) inhibitors (secukinumab, ixekizumab and ustekinumab) are currently approved in Italy for the treatment of both PsO and PsA, while other interleukin inhibitors (guselkumab, tildrakizumab, brodalumab and risankizumab) are specifically approved for PsO, and abatacept, golimumab and certolizumab pegol are only approved for PsA treatment. Apremilast, a targeted synthetic Disease-Modifying anti-rheumatic drug (tsDMARD), is also approved for the treatment of adult patients with PsA who have had an inadequate response or who have been intolerant to a prior DMARD therapy, and for patients with moderate to severe chronic PsO who failed to respond to, who have a contraindication to, or who are intolerant to other systemic therapy, including cyclosporine, MTX or PUVA [16].

Despite the wide range of therapeutic options, recent US real world studies among PsO/PsA patients using questionnaire-based surveys, chart reviews and claims data have identified potential undertreatment and treatment dissatisfaction as significant barriers to optimal disease care [17,18,19,20,21]. Data from a Belgian cross-sectional study confirmed that undertreatment represents a challenge in PsO management: the results reported that almost 40% of patients with moderate-to-severe PsO were not treated with any systemic therapy (or with any therapy at all, including topical drugs) despite the disease severity [22].

To our knowledge, population-based drug utilization studies in PsO and PsA patients in real-world settings are scarce in Italy. This study was aimed at exploring the pattern of use of topical and various systemic pharmacological treatments in PsO and PsA patients from a large local health unit of Southern Italy during the years 2016–2018, with the specific goal to explore whether those patients are undertreated with systemic drugs.

## 2. Materials and Methods

### 2.1. Data Source

A retrospective cohort study was performed. Fully anonymized data were extracted from the claims database of the Caserta Local Health Unit (LHU), covering a total population of almost 1 million persons during the years 2010–2018. The collected data included: (1) a demographic database, including information about the date of birth, gender, date of registration in the regional healthcare system and, where applicable, the date and cause of death, coded with International Classification of Diseases, ninth revision, clinical modification (ICD9-CM); (2) an out-patient pharmacy database, including data on the date of the drug dispensed, the number of dispensed packages and the active substance and brand name, coded with the Anatomical Therapeutic Chemical classification system (ATC) and AIC (i.e., Italian market authorization) codes; (3) a hospital discharge database, containing information on the date of hospital admission and discharge, diagnosis-related group (DRG), principal diagnosis and up to five secondary diagnoses, and principal procedure and up to five secondary procedures, coded with ICD-9-CM codes; (4) requests for outpatient diagnostic tests and the specialist visits database, including test- or visit-specific codes, the date of the test and the name of the laboratory where the test was carried out; (5) an exemptions from healthcare service co-payment database, which contains coded information about chronic diseases or socioeconomic factors. In addition, electronic therapeutic plans (for bDMARDs and tsDMARDs only), including information on the prescribed dosing regimen, indications for use and length of therapy were available.

All of the databases were linked through a unique anonymized patient identifier with each other and with the medical records of general practitioners’ (GPs) practicing in the same catchment area, and collecting information on the indications for use of all of the prescribed drugs. Italy has a universal healthcare system, where all of the National Health System (NHS) beneficiaries (i.e., all of the residents in any given catchment area) are registered in a demographic database. All hospitalizations are fully reimbursed by the Italian NHS, while outpatient diagnostic tests and specialist visits are almost completely reimbursed by the NHS as well, unless patients decide to access private healthcare services. Concerning drugs, almost 80% of all drugs are fully reimbursed by the NHS (including biological drugs), with the remainder being in the charge of citizens (e.g., over the counter drugs). All of these claims are traced in this study.

Caserta record linkage databases have been shown to provide accurate and reliable information for pharmacoepidemiology research, as documented elsewhere [23,24,25].

### 2.2. Study Population

During the years 2010–2018, all of the subjects registered in the Caserta LHU database with at least a PsO/PsA diagnosis were included in the study. The diagnoses of PsO or PsA were identified by searching for: (a) specific ICD-9 CM codes (i.e., 696.1 for PsO and 696.0/713.3 for PsA) from discharge diagnosis/indications for use from electronic therapeutic plans/GP medical records; and/or (b) exemption from healthcare service co-payment codes (i.e., 045.696.1 for PsO and 045.696.0 or 045 + certolizumab pegol/golimumab dispensing for PsA) during the period 2010–2018. Because certolizumab pegol and golimumab are biological drugs approved for PsA, as well as other immune-mediated inflammatory diseases (e.g., rheumatoid arthritis, spondyloarthritis), the association with the “045” generic exemption code was considered as a proxy of PsA. The date of first diagnosis of PsO/PsA was defined as the index date (ID).

### 2.3. Study Follow-Up

The follow-up period for each PsO/PsA patient started from the ID, and was censored with the occurrence of one of the following events, whichever came first: (a) the patient’s death; (b) transfer out of the database; or (c) the end of the observation period (31 December 2018).

### 2.4. Exposure Assessment

All of the following drug classes were included: non-DMARDs (i.e., antipsoriatics for topic use, antipsoriatics for systemic use, topical and systemic glucocorticoids, NSAIDs), csDMARDs (i.e., methotrexate, cyclosporine, leflunomide, sulfasalazine), targeted synthetic DMARDs (tsDMARDs) (i.e., apremilast); bDMARDs, both originators and biosimilars, belonging to different mechanistic classes such as TNF-alpha inhibitors (adalimumab, etanercept, infliximab, golimumab, certolizumab pegol), selective immunosuppressants (abatacept) and IL-inhibitors (ixekizumab, secukinumab, ustekinumab, guselkumab). As they were marketed after the end of the study period, guselkumab, tildrakizumab, brodalumab and risankizumab were not included in this analysis. The different therapy lines and eligibility criteria for biological treatment in PsO and PsA patients are described in detail in the Supplementary Materials.

### 2.5. Data Analyses

The prevalence of PsO and PsA was measured by searching the above described criteria during the period 2010–2018. Specifically, the number of subjects with at least one day of database history in Caserta LHU during the years 2016–2018, and with PsO/PsA diagnosis registered anywhere from 1 January 2010 to 31 December 2018 was considered as the numerator, while the number of residents in the catchment area during the years 2016–2018 was considered as the denominator.

The PsO/PsA patients were characterized in terms of sex and mean age (±Standard Deviation, SD).

The proportion of PsO/PsA patients either receiving, respectively, at least one dispensing of non-DMARDs, csDMARDs, bDMARDs, tsDMARDs or csDMARDs + bDMARDs, or not receiving any of the study drugs included in the above mentioned classes from the ID onwards was calculated in the period 2016–2018. Patients were included in the analysis if they had at least 6 months of continuous enrollment after ID in the database in order to ensure the comprehensive assessment of the provided healthcare services, including drugs. Specifically, the patients were grouped based on the dispensed study drugs from the ID onwards, in the following mutually exclusive categories: (a) non-DMARD users, if they received at least one non-DMARD dispensing and no dispensing of bDMARDs/csDMARDs/tsDMARDs; (b) csDMARD users, if they received at least one csDMARD dispensing and no dispensing of bDMARDs/tsDMARDs (irrespective of non-DMARD dispensing); (c) bDMARD users if they received at least one bDMARD dispensing (irrespective of non-DMARD/csDMARD/tsDMARD dispensing); (d) tsDMARD users if they received at least one tsDMARD dispensing (irrespective of non-DMARD/csDMARD/bDMARD dispensing); (e) csDMARD + bDMARD users if they received at least one csDMARD and at least one bDMARD dispensing; (f) untreated patients, if they did not receive any dispensing of the study drugs. The frequency of PsO or PsA patients with at least 6 months post-ID continuous enrollment in the database was calculated. The analyses were stratified by drug classes and active substances during the years 2016–2018.

Among the PsO/PsA patients with at least one bDMARD dispensing in the study period, the proportion of subjects with at least one diagnosis of other immune-mediated inflammatory diseases for which bDMARD is also approved (e.g., inflammatory bowel disease -IBD (Crohn’s disease and ulcerative colitis), uveitis, hidradenitis suppurativa) any time prior to the first bDMARD dispensing, was calculated. For each study patient with at least one bDMARD dispensing, the median time (months) from the first PsO/PsA diagnosis to the first bDMARD dispensing, and the median time (months) from the first csDMARD dispensing to the first bDMARD dispensing were calculated by excluding from these analyses those patients with concomitantly immune-mediated inflammatory disease diagnoses other than PsO/PsA. The time from the first registered disease diagnosis/first csDMARD dispensing to the first bDMARD dispensing was assessed using a Kaplan–Meier plot, and was stratified by indication for use.

All of the analyses and plots were performed using SAS software, Release 9.4 (SAS Institute, Cary, NC, USA), and ERRE software, version 4.0.3.

## 3. Results

**Figure 1:**
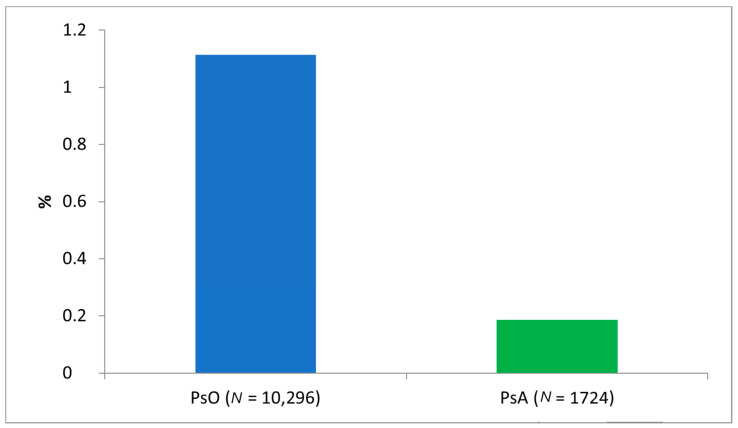
Prevalence (%) of patients with psoriasis and psoriatic arthritis in Caserta Local Health Unit in the years 2010–2018. Legend: Pso, psoriasis; PsA, psoriatic arthritis.

During the study period, 10,296 subjects with a diagnosis of PsO and 1724 subjects with diagnosis of PsA were identified from Caserta LHU (Figure 1), yielding a prevalence of 1.1% and 0.2% for PsO and PsA, respectively. The mean age was 51.5 in both cohorts. Specifically, only 276 (2.7% of the total PsO patients) PsO patients (mean age ± SD: 14.2 ± 3.4) and 30 (1.7%) PsA patients (mean age ± SD: 14.0 ± 3.9) aged less than 18 years old were identified. Significant sex differences were observed between the two cohorts (*p*-value < 0.0001): the M/F ratio favoured females in the cohort of PsA patients (M/F ratio = 0.76), while no gender differences were reported in the group of PsO patients (M/F ratio = 1.03).

**Figure 2:**
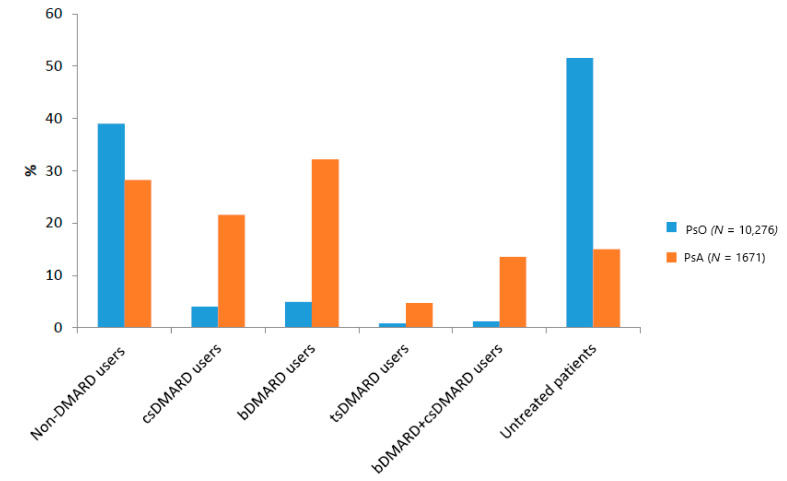
Proportion of pharmacological treatment lines in PsO and PsA patients in Caserta LHU during the years 2016–2018. Legend: bDMARD, Biological Disease-Modifying Anti-rheumatic Drugs; csDMARD, Conventional Disease-Modifying Anti-rheumatic Drugs; PsA, psoriatic arthritis; Pso, psoriasis; tsDMARD, Targeted Disease-Modifying Anti-rheumatic Drugs. Note: Only PsO and PsA patients with at least 6 months post-Index continuous enrollment in their database were included in this analysis.

During the period 2016–2018, more than half of the psoriatic patients (*N* = 5301; 51.6%) were untreated, compared to 251 (15%) of the PsA patients (Figure 2).

**Figure 3:**
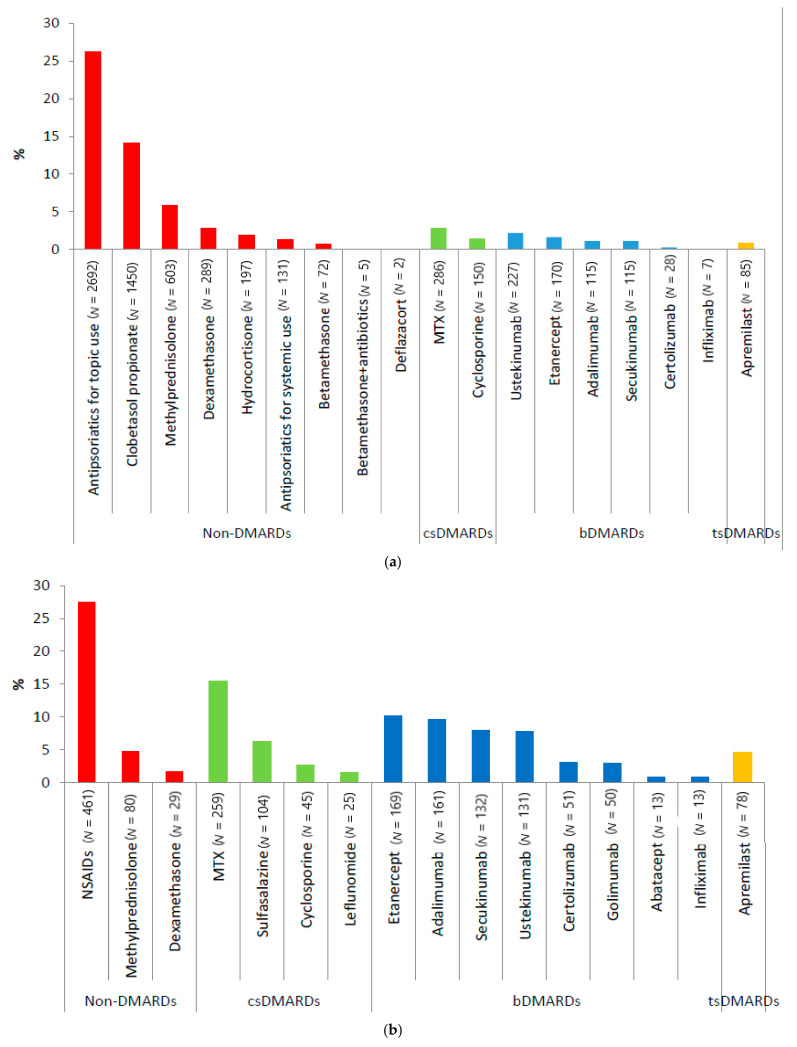
Distribution (%) of different compounds by pharmacological treatment lines in PsO (**a**) and PsA (**b**) patients in the Caserta LHU during the years 2016–2018. Legend: bDMARD, Biological Disease-Modifying Anti-rheumatic Drugs; csDMARD, Conventional synthetic Disease-Modifying Anti-rheumatic Drugs; DMARD: Disease-Modifying Anti-rheumatic Drugs; MTX, methotrexate; NSAIDs, nonsteroidal anti-inflammatory drugs; PsA, psoriatic arthritis; PsO, psoriasis; tsDMARD: Targeted Synthetic Disease-Modifying Anti-rheumatic Drugs. Note: Only PsO/PsA patients with at least 6 months post-Index continuous enrollment in their database were included in this analysis.

Regarding those treated, 4009 PsO patients (39.0%) were only treated with non-DMARDs, mostly antipsoriatics for topical use (26.2%). Less than 5% of the PsO patients received either csDMARDs or bDMARDs (mostly ustekinumab), and a very low proportion of them were treated with both drug classes (*N* = 121; 1.2%) (Figure 2 and Figure 3a). In contrast to the PsO patients, a larger proportion of PsA patients were treated with bDMARDs (*N* = 538; 32.2%) (mostly with etanercept and adalimumab) or with non-DMARDs only (*N* = 472; 28.2%), followed by csDMARDs (*N* = 360; 21.5%) (Figure 2 and Figure 3b). Among the non-DMARDs with indication for PsA, NSAIDs were the most frequently dispensed drugs. TsDMARD (i.e., apremilast) was rarely used in both diseases, with a slightly higher percentage in PsA than PsO patients (4.7% vs. 0.8%) (Figure 3).

Among the PsO patients treated with bDMARDs, 181 (35.9%) had a history of PsA, 70 (13.9%) had a history of IBD, and 63 (12.5%) had a history of uveitis. Among the PsA patients treated with bDMARDs, 254 (47.2%) subjects had a history of PsO, followed by 128 (23.8%) with IBD and 122 (22.7%) with uveitis (data not shown).

**Figure 4:**
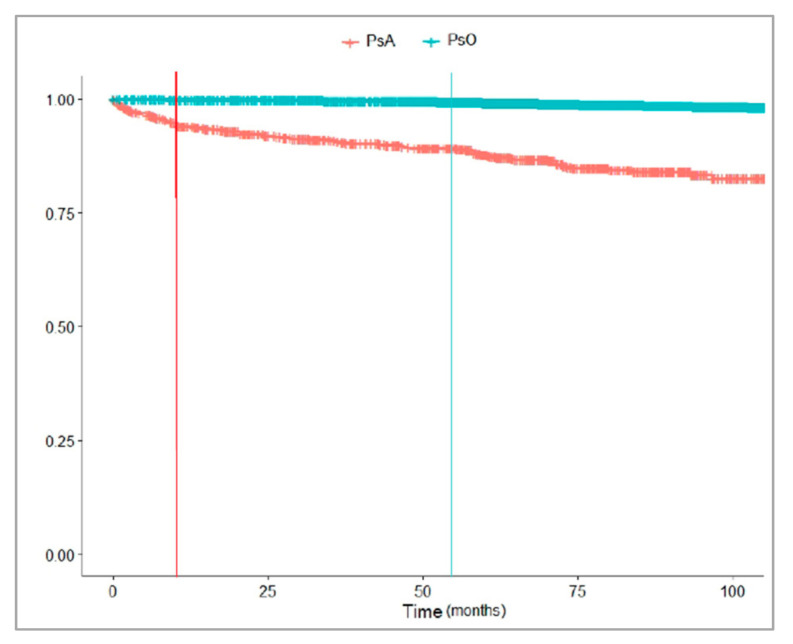
Time (months) from the first registered PsO/PsA diagnosis to the first bDMARD dispensing in the years 2010–2018. Legend: PsA, psoriatic arthritis; PsO, psoriasis.

**Figure 5:**
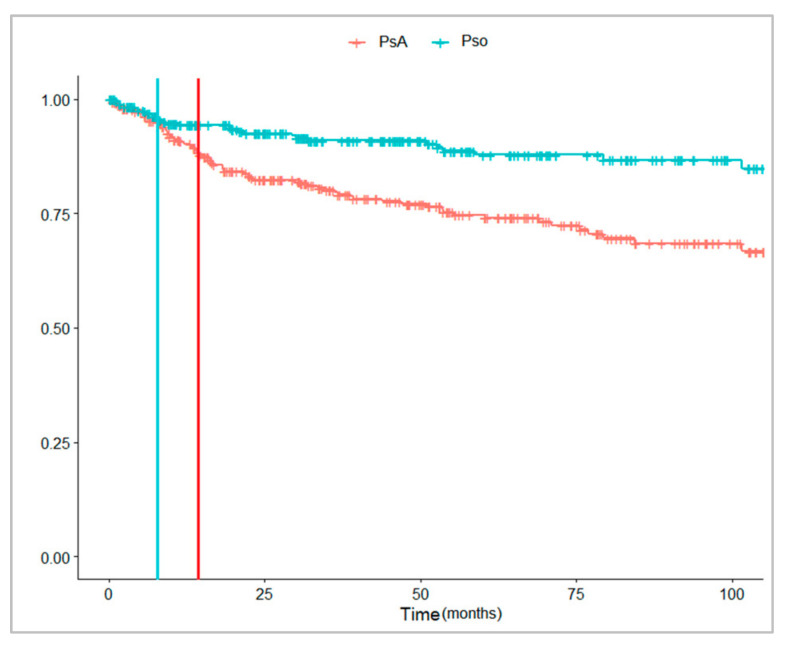
Time (months) from the first csDMARD dispensing to the first bDMARD dispensing in the years 2010–2018, stratified by PsA/PsO. Legend: PsA, psoriatic arthritis; PsO, psoriasis.

Figure 4 and Figure 5 showed that a lower proportion of PsO than PsA patients received a bDMARD during the follow-up. The median time from the first registered disease diagnosis to the first bDMARD dispensing was shorter in the patients with PsA [13.3 months (Q1–Q3: 3.1–43.9)] than in those with PsO [54.2 months (Q1–Q3: 30.5–72.2)]. Instead, the median time from the first csDMARD dispensing to the first bDMARD dispensing was shorter in PsO [9.2 months (Q1–Q3: 5.5–30.0)] than in PsA patients [14.5 months (Q1–Q3: 8.6–33.5)].

## 4. Discussion

This large retrospective cohort study investigated the pattern of use of different pharmacological treatment lines (non-DMARDs, csDMARDs, tsDMARDs and bDMARDs) in PsO and PsA patients from a general population in Southern Italy, with a focus on the appropriate access to the most recently marketed drugs.

Our data suggest that the prevalence of PsO was lower than the prevalence reported in previous Italian studies (1.1% vs. 2.7%) [2,4], but it was almost in line with the prevalence reported in previous studies using the claims database (1.5%) [26]. Instead, PsA’s estimated prevalence of 0.2% is in line with previous studies, based on the claims database as well as other data sources (0.1–0.2%) [27,28].

Of the patients who received prescription therapy, most of PsO patients received only non-DMARDs (almost 40%), with antipsoriatics for topical use being the most frequently prescribed treatment in those patients (26.2%). It is known that most psoriatic patients have mild psoriasis [29], for which topical drugs (mainly corticosteroids) represent the mainstay therapy.

A higher proportion of PsA patients (21.5%) than PsO patients (4.0%) were treated with csDMARDs. Interestingly, although MTX and leflunomide are the only conventional systemic drug therapies approved in Italy for the PsA treatment, we found that PsA patients were also treated with other csDMARDs (i.e., sulfasalazine and cyclosporine), with no indication for use in the summary of the product characteristics (SmPCs) for PsA [16]. According to Italian SmPC, sulfasalazine could also be used for concomitant immune-mediated inflammatory diseases (i.e., inflammatory bowel diseases as well as rheumatoid arthritis); however, its use in PsA patients is also recommended by therapeutic guidelines [14,15].

A possible explanation for the cyclosporine use in PsA patients is the concomitant severe psoriasis for which cyclosporine is indicated.

Our results confirm previous research [18,19,22,30] that identified the lack of treatment as a substantial problem for patients with PsO and PsA. In our study, more than 50% of PsO patients and 15% of PsA patients were not treated with prescribed drugs during the 2 year observation period. An exploratory analysis showed that 22.6% and 21.9% of the untreated PsO and PsA patients received colecalciferol dispensing, probably to treat the vitamin D deficiency related to the study diseases. Moreover, among the untreated PsO and PsA patients, 3.7% and 7.2% PsO and PsA patients, respectively, were aged less than 18 years old. In general, the proportion of untreated patients was difficult to compare with previous studies that were only restricted to patients with moderate to severe diseases; two U.S. questionnaires-based surveys reported a comparable proportion of PsO and PsA patients (irrespective of disease severity) compared to our study [18,19]. The National Psoriasis Foundation Surveys reported 24–35% of untreated patients with moderate PsO, and 9–30% of untreated patients with severe PsO [18]. Previous surveys also indicated that a large proportion of patients with psoriasis are frequently undertreated or unsatisfied with their current treatment. In particular, a cross-sectional observational study conducted in Belgian dermatology centers in 2011–2012 reported that 38.1% of psoriatic patients were not treated with any systemic drugs. Furthermore, the results from a survey conducted in the U.S. showed that—among PsO patient responders—39% of patients with severe disease and 37% with moderate disease were not receiving any treatment [22,30]. Although we were unable to grade the PsO and PsA severity, due to lack of these clinical information in the claims database, the high percentages of untreated patients shown in our study point overall towards a potential undertreatment, and require further investigation.

A higher proportion of the PsA patients (32.2%) were treated with bDMARDs (mostly with etanercept and adalimumab) compared to the PsO patients (4.9%), in which ustekinumab was the most frequently used bDMARD. Compared with a previous drug utilization study conducted in Italian PsO and PsA patients [31], etanercept, adalimumab and ustekinumab were the three most frequently reported bDMARDs as the first biological treatment; however, the proportion of PsO and PsA patients treated with these compounds was higher, except for ustekinumab in PsA, compared to our study. Furthermore, Marcianò et al. documented, using the claims database, an underutilization of bDMARDs in PsO/PsA patients from another general population of Southern Italy in the years 2010–2014 [32,33].

Based on the Italian SmPCs, biological drugs represent a line of therapy usually reserved for patients who have failed or have contraindications to csDMARDs (Supplementary Materials). However, it has been reported that in patients with severe clinical conditions (e.g., severe psoriasis and active PsA) and with a higher risk of cardiovascular diseases (e.g., obesity, hypertension, diabetes or dyslipidemia), bDMARDs, particularly TNF-alpha inhibitors, may be prescribed at an earlier stage, as these drugs may reduce the inflammatory burden and should be particularly preferred to cyclosporine (which may worsen hypertension, dyslipidemia, diabetes or renal disease) or acitretin (which can induce or worsen dyslipidemia) in patients at cardiovascular risk [34]. The prescription of bDMARDS in PsO and PsA patients may also be influenced by the presence of contraindications (e.g., infections) to the treatment, or loco-regional restrictions for cost containment. In line with these considerations, two surveys conducted in the U.S. among clinicians reported that the common barriers for dermatologists and rheumatologists to start bDMARDs therapy in moderate-to-severe disease patients included uncertainty over long-term safety, contraindications and high costs [20,35].

It is known that approximately 30% of patients with PsO will develop PsA [5,6,36], as we observed in our study showing the proportion of biological drug users in patients with other immune-mediated inflammatory diseases. Unexpectedly, among the PsA patients, only 47.2% of bDMARDs users also had psoriasis. In a recent review, Armstrong et al. reported that in almost 85% of patients with psoriatic arthritis, psoriasis either precedes or occurs concurrently with psoriatic arthritis [37]. The underestimation coming from our data should be explained by a lack of information in the coding of a concurrent diagnosis of cutaneous psoriasis by some rheumatologists.

The median time from the diagnosis to the first bDMARDs and the median time from the first csDMARD to the first bDMARD were assessed and stratified by the indications for use. The median time from diagnosis to bDMARDs in the PsO patients was 54.2 months (Q1–Q3: 30.5–72.2), which was not in line with previous research conducted in the U.S. that reported a median time of 196 days (6.5 months) [38]. This difference probably reflects the different underlying national healthcare system and the attitude to the access to more innovative and high costs of bDMARDs in the USA than Italy.

This study has some limitations that warrant caution. First of all, the clinical data about PsO and PsA patients such as the PASI (i.e., the score related to the PsO severity) were missing in the databases; as such, it was not possible to evaluate the specific level of disease severity with the pattern of drug use. However, by identifying the PsO patients based on the hospital discharge database, exemptions or biological drug dispensing, we could assume in our cohorts a large proportion of patients with moderate–severe PsO and/or PsA.

Second, some study drug dispensing might not have been captured by the LHU databases (e.g., phototherapy, topical therapies or NSAIDs that are not reimbursed by the National Health System). In particular, the low traceability of the phototherapy, topical therapies or NSAIDs could lead to a potential overestimation of the undertreated PsO patients.

Finally, our research is based on a restricted Italian geographic area, and may not be representative of the entire Italian population.

## 5. Conclusions

Our findings seems to suggest a potential undertreatment of PsO (much less for PsA) in an LHU of Southern Italy, with a particularly low use of the more recently marketed drugs, such as biological ones. The reasons for this should be further explored in future studies, collaboratively involving patients as well as clinicians.

## Data Availability

Fully anonymized dataset is available only upon request from the corresponding author, as there is an agreement between the University of Messina and the data provider (Caserta Local Health Unit) not to share the data publicly.

## Supplementary Materials

The following are available online at https://www.mdpi.com/article/10.3390/jcm10153431/s1. Table S1: Different therapy lines and eligibility criteria to biologic treatment in patients with psoriasis or psoriatic arthritis.
