# Supplementary material for: Are Patients with Psoriasis and Psoriatic Arthritis Undertreated? A Population-Based Study from Southern Italy
Source: J Clin Med. 2021 Jul 31;10(15):3431. doi: 10.3390/jcm10153431 (PMC8348176; doi:10.3390/jcm10153431)
Supplement: Supplementary file 1 [file jcm-10-03431-s001.zip › jcm-1276922-SI.pdf]

Journal of
Clinical Medicine

MDPI

Table S1. Different therapy lines and eligibility criteria to biologic treatment in patients with psoriasis or psoriatic arthritis.

Disease	Therapy Lines			Eligibility Criteria to Biologic Treatment
	I Line	II Line	III Line	
Psoriasis	Mild psoriasis:<br><br>Topical corticosteroids<br>• Betamethasone/antibiotics fixed combination<br>• Dexamethasone/antibiotics fixed combination<br>• Betamethasone<br>• Clobetasol propionate<br>• Dexamethasone<br>• Desonide<br>• Hydrocortisone<br>• Methylprednisolone<br>• Mometasone furoate<br>• Triamcinolone<br><br>Systemic glucocorticoid:<br>• Dexamethasone i.v.<br><br>Other drugs used in psoriasis (topic administration):<br>• Calcipotriol<br>• Calcipotriol/Betamethasone fixed combination<br>• Calcitriol<br>• Tacalcitol<br>• Tazarotene<br>• Dithranol<br>• Methoxsalen	Psoriasis moderate-severe grade[11,16]:<br><br>**DMARDs:**<br>• MTX;<br>• Cyclosporine (severe psoriasis in patients after failure of conventional therapy or when it was not appropriate<br><br>Systemic glucocorticoid:<br>• Deflazacort<br><br>Antipsoriatics for systemic use<br>• Acitretin<br><br>Other antipsoriatic treatments:<br>• PUVA	**bDMARDs[11,16]:**<br>• Secukinumab<br>• Ixekizumab<br>• Adalimumab (40-80 mg)<br>• Infliximab<br>• Certolizumab<br>• Etanercept<br>• Ustekinumab<br>• Brodalumab<br>• Risankizumab<br>• Tildrakizumab<br>• Guselkumab<br><br>**tsDMARDs[11,16]**<br>• Apremilast	• Inadequate response, controindications or intolerance to other sistemic treatments (e.g. cyclosporine, MTX or PUVA) (Infliximab, Etanercept, Ustekinumab)<br><br>• Adult patients with moderate or severe plaque psoriasis who were candidates for systemic therapy (Secukinumab, Ixekizumab, Adalimumab)<br><br>Comorbities that could be treated with the same drug (e.g. psoriatic arthritis, Crohn's disease, ulcerative colitis, uveitis)[11]
Psoriatic arthritis	**csDMARDs:**<br>• Leflunomide[16]<br>• MTX[16]<br>• Sulfasalazine and Cyclosporine (according to guidelines, but with no indication for psoriatic arthritis in SmPCs[16,39]	**bDMARDs[16]:**<br>• Abatacept<br>• Adalimumab (40 mg)<br>• Certolizumab pegol<br>• Etanercept<br>• Golimumab<br>• Infliximab	**tsDMARDs[16,40]:**<br>• Apremilast monotherapy or + csDMARDs, in adult patients who have had an inadequate response or who have been intolerant	• Failure or inadequate response at least to a cDMARDs as monotherapy or as fixed combination for 3 months[39,41] (bDMARD as monotherapy or +MTX)[16]

**NSAIDs**[40]<br><br>**Glucocorticoids**[16]**:**<br>• Deflazacort<br>• Dexamethasone (additional therapy for a short period of time as relief of an acute episode or exacerbation)<br>• Methylprednisolone acetate (suspension for injection)	• Ixekizumab<br>• Secukinumab<br>• Ustekinumab	to a prior DMARD therapy	• Patients for whom additional systemic therapy for psoriatic skin lesions is not required (Abatacept)<br><br>Severe active disease (≥5 swollen joints and high inflammatory indexes) and aggressive (e.g. anatomical joint damage)[39]

Legend: bDMARDs: Biological Disease Modifying Anti-rheumatic Drugs; csDMARDs: Conventional Synthetic Disease Modifying Anti-rheumatic Drugs; MTX: methotrexate; NSAIDs: Non-steroidal anti-inflammatory drugs; NYHA: New York Heart Association; PUVA: psoralen and ultraviolet A; SmPCs : summary of product characteristics; tsDMARDs: Targeted Synthetic Disease Modifying Anti-rheumatic Drugs.
